# An Automated, Home-Cage, Video Monitoring-based Mouse Frailty Index Detects Age-associated Morbidity in C57BL/6 and Diversity Outbred Mice

**DOI:** 10.1093/gerona/glad035

**Published:** 2023-01-27

**Authors:** J Graham Ruby, Andrea Di Francesco, Paulo Ylagan, Angela Luo, Robert Keyser, Owen Williams, Sarah Spock, Wenzhou Li, Nalien Vongtharangsy, Sandip Chatterjee, Cricket A Sloan, Charles Ledogar, Veronica Kuiper, Janessa Kite, Marcelo Cosino, Paulyn Cha, Eleanor M Karlsson

**Affiliations:** Calico Life Sciences LLC, South San Francisco, California, USA; Calico Life Sciences LLC, South San Francisco, California, USA; Calico Life Sciences LLC, South San Francisco, California, USA; Calico Life Sciences LLC, South San Francisco, California, USA; Calico Life Sciences LLC, South San Francisco, California, USA; Calico Life Sciences LLC, South San Francisco, California, USA; Calico Life Sciences LLC, South San Francisco, California, USA; Calico Life Sciences LLC, South San Francisco, California, USA; Calico Life Sciences LLC, South San Francisco, California, USA; Calico Life Sciences LLC, South San Francisco, California, USA; Calico Life Sciences LLC, South San Francisco, California, USA; Calico Life Sciences LLC, South San Francisco, California, USA; Calico Life Sciences LLC, South San Francisco, California, USA; Calico Life Sciences LLC, South San Francisco, California, USA; Calico Life Sciences LLC, South San Francisco, California, USA; Calico Life Sciences LLC, South San Francisco, California, USA; Calico Life Sciences LLC, South San Francisco, California, USA

**Keywords:** Age-related pathology, Bioinformatics, Digital biomarkers, Frailty, Home cage, 3Rs (reduce replace refine)

## Abstract

Frailty indexes (FIs) provide quantitative measurements of nonspecific health decline and are particularly useful as longitudinal monitors of morbidity in aging studies. For mouse studies, frailty assessments can be taken noninvasively, but they require handling and direct observation that is labor-intensive to the scientist and stress inducing to the animal. Here, we implement, evaluate, and provide a refined digital FI composed entirely of computational analyses of home-cage video and compare it to manually obtained frailty scores in both C57BL/6 and genetically heterogeneous Diversity Outbred mice. We show that the frailty scores assigned by our digital index correlate with both manually obtained frailty scores and chronological age. Thus, we provide an automated tool for frailty assessment that can be collected reproducibly, at scale, without substantial labor cost.

For many organisms, increasing age for an individual is accompanied by physiological deterioration and an increase in mortality hazard. At a population level, physiological decline with age can be measured using mortality statistics, and for many species, the risk of death increases exponentially with age (1). While useful for performing comparisons between species or populations, mortality provides only one datum per individual and therefore requires large cohorts and precludes longitudinal analysis of individuals.

In humans, the physiological decline that accompanies age can also be measured in terms of a “frailty index” (FI): a tally of health-related deficits that are clinically observable (2). Many varieties of human FI exist, with variable focus on performance-based phenotypic assessment (3) or the accumulation of overt pathologies (eg, (4)) but consistently predict the likelihood of future disability and mortality (eg, (5)). Due to their implicitly multifaceted nature, frailty assessments often require substantial time, cost, and expertise to administer. Many variants of human FI implementation attempt to reduce those practical constraints: among them, a version requiring minimal and non-expert examination (6); a version derived exclusively from blood-test measurements (7); and a version based solely on retrospective analysis of electronic health records (8).

The FI concept is also applied to mice in preclinical research (9,10). As with humans, alternative versions of a mouse FI have been implemented that reflect diverging priorities: some emphasize thoroughness and include laboratory tests (eg, (11)); others attempt to maximize similarity to human FI (12). Most FIs involve subjective scoring by a trained researcher, which poses 2 challenges to the practical application of FI. First, the multitude of traits to be examined places a substantial time demand on the researcher. Second, while FI rater agreement is high under ideal conditions, in practice, human factors such as insufficient communication or uneven educational background can result in biases that reduce inter-rater reproducibility (13–15).

Consistency of mouse FI across raters has been mitigated through automation of analysis. For grimace—a common component of manual FIs that indicates discomfort (16) —machine-learning (ML) assessments perform similarly to human raters when applied to images captured in tabletop cubicles (17). Parameters derived from the open-field assay (18), for which behavior is often analyzed using computer vision (19), are often included in traditional frailty assessments (eg, (9)). The convenience of the open-field paradigm for analysis of multiple traits has motivated the development of mouse FI versions that rely entirely on data from that assay (20). In addition to improved reliability, these cases greatly ease the practical application of FI by consolidating data collection to a single, low-labor assay.

The approaches to mouse frailty assessment vary in terms of their invasiveness, but they invariantly require extensive handling and/or exposure to a non-standard environment to be performed. Handling and exposure to novel environments both cause stress in laboratory mice (21–23), with even routine husbandry procedures driving increased activity (24) and corticosterone levels (25). The FIs described above invariantly include parameters known to be modified by handling-related stress: posture, response to stimuli, exploratory behavior, and blood chemistry among them (26–28). The recent development of an ML-based FI in the context of an open-field environment (20) represents a major advance in this regard, greatly minimizing the handling of animals—though still monitoring the mice in a stressful, brightly lit environment (29). The development of systems capable of continuously monitoring mice in their home-cage environment (30,31) provides an avenue to increase throughput and refine longitudinal aging studies while minimizing handling-related stress as confounders of frailty assessment. But, that advancement requires the development of analytics capable of measuring frailty from home-cage video data, akin to those developed for a video-captured open-field environment (20).

Here, we implemented a digital frailty index (DFI) for mice based on the computational analysis of continuously collected home-cage video. To evaluate its effectiveness, we performed 2 studies: the first was small scale, using male C57BL/6J mice; the second was large scale, including over 200 Diversity Outbred (J:DO) mice, both females and males. In both cases, video was collected in parallel with manual frailty indexes (MFI) across mice of a variety of ages. Our implementation of DFI correlated with both chronological age and MFI, the correlation between DFI and MFI was maintained when the age-related components of both measurements were regressed out. While the inclusion of additional parameters may enhance DFI value in the future, here, we prove the feasibility, scalability, and relevance of a refined frailty assessment in mice through noninvasive observation in a home-cage environment.

## Method

### Mice, Animal Husbandry, and In Vivo Study Design

For the preliminary study, 31 male C57BL/6J mice (The Jackson Laboratory, Bar Harbor, ME, Strain #000664) were obtained from The Jackson Laboratory at 3 months of age, then aged in group housing (3–5 mice/cage) at Calico until the beginning of MFI and DFI measurements. At the beginning of the study period, these mice weighed between 26 g and 46 g. Manual frailty assessments were performed immediately prior to the onset of video collection, which continued for 20 days. Mice were singly-housed during video collection. Census data on these mice are provided in Supplementary Table 1. Housing details are provided in the Supplementary Text.

For the J:DO study, 228 Diversity Outbred (J:DO) mice (138 female, 90 male; The Jackson Laboratory, Strain #009376) across 8 aged cohorts were obtained from The Jackson Laboratory. Mice were aged to 6, 9, 12, 15, 19, 21, 25, 27, and 30 months at the beginning of the study period and weighed between 18 g and 67 g. Video collection and manual frailty assessments were performed at 3 time points, 6 weeks apart. Females were group housed when not in video cages, and males were singly housed throughout the study. Mice were shipped from The Jackson Laboratory and acclimated for at least 2 weeks. Census data on these mice are provided in Supplementary Table 2. Housing details are provided in the Supplementary Text. A schematic of the experiential design is provided as Supplementary Figure S1.

For the C57B/6J and J:DO studies, manual frailty was assessed as described in the original 31-item mouse clinical FI (10), omitting 2 parameters reliant on population-specific statistics (see Supplementary Methods). Mice were allowed to acclimatize to the testing room for 30–45 minutes before testing. Observations were carried out on an open bench at the same time of the day, between 9 and 11 am. Mice were scored 0, 0.5, or 1 based on the severity of deficit they showed in each of the 29 items, with 0 representing no sign of deficit, 0.5 mild deficit and 1 severe deficit. The overall MFI was taken as the average score across the 29 included parameters. These data are provided in Supplementary Table 1 for the C56B/6J study and Supplementary Table 3 for the J:DO study.

**Figure 1.**
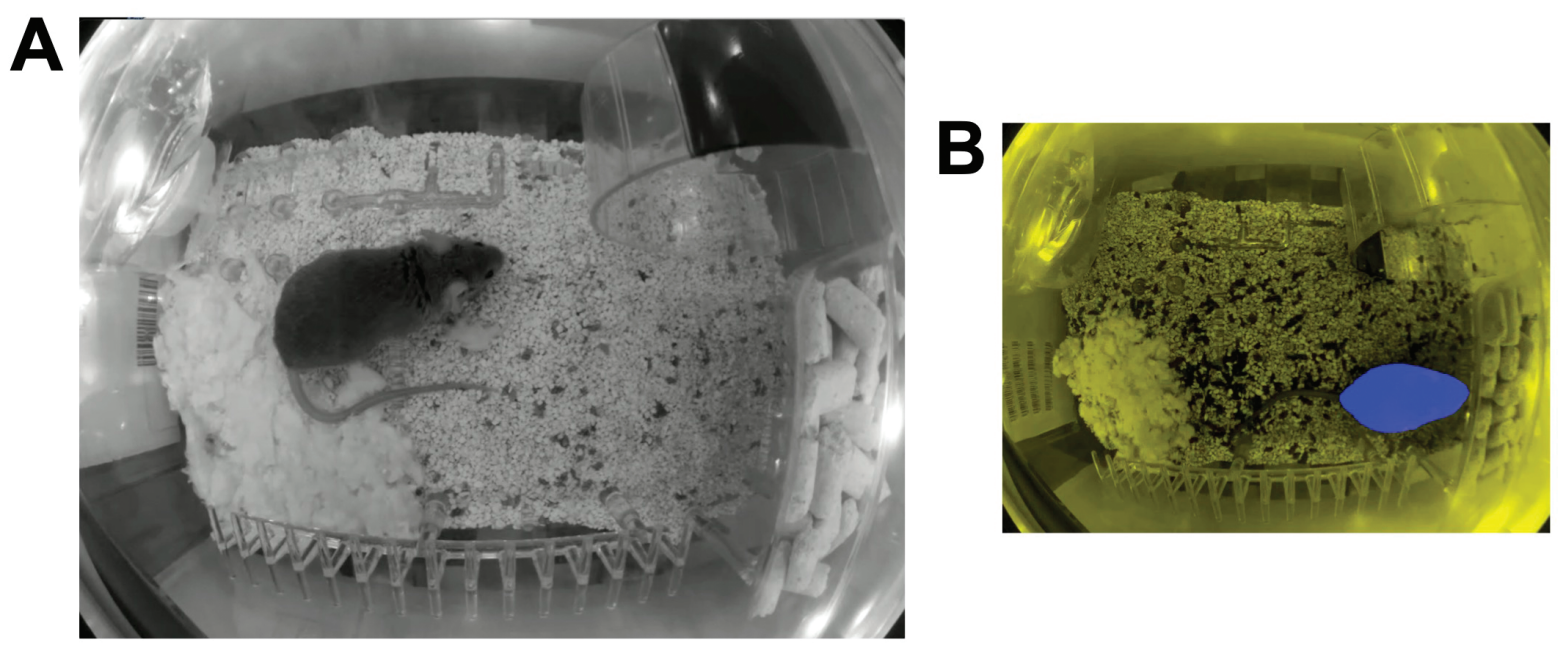
Example video stills and methodological details. (**A**) An example frame of video footage, typical of nighttime video collected in this study. Nighttime illumination was near-IR, resulting in grayscale images. Daytime images were similar, but in RGB color. (**B**) An example of a modified input image for body-weight prediction error-detection models, with the blue channel modified to indicate masked (blue) versus unmasked (yellow) output from the mouse semantic segmentation model. See Method for details.

Video was collected by placing cages with singly-housed mice into video camera-equipped racks for approximately 1 week. Video footage of mouse cages was continuously streamed to a cloud-based data infrastructure. Video acquisition hardware and compatible cage furniture were purchased from Vium (https://www.vium.com). Data management software was created by the authors of this study. All videos were collected at 864 pixel (width) by 648 pixel (height) resolution, approximately 24 frames per second, from cameras mounted in consistently fixed positions versus the cage/furniture defined by the architecture of the Vium racks (eg, Figure 1A).

Video data for model training and parameterization were collected separately, under similar conditions as for the main 2 experimental studies. Those data included J:DO mice, aged to 19 months (*n* = 40), 26 months (*n* = 37), and 32 months (*n* = 31) and monitored for one week; and C57BL/6J mice, aged to 7, 19, and 35 months (*n* = 10 per age cohort) and monitored for 3 weeks.

All research was performed as part of Calico Life Sciences LLC (South San Francisco, CA) AAALAC-accredited animal care and use program. All research and animal use in this study was approved by the Calico Institutional Animal Care and Use Committee.

### Calculation of Digital Frailty Index score

Below, we describe the methods that were used to analyze raw video data and arrive at a DFI score. Additional details for some methods are provided in the Methods section of the Supplementary Text. Further, the entire code base and accompanying ML models are available at https://github.com/graham-calico/DFI_v1.

Eight frailty parameters were scored: average per-day distance run on the wheel; gait speed on the wheel; gait speed on the floor of the cage; circadian distribution of wheel-running activity; circadian distribution of movement on the floor of the cage; rate of change in body weight; coat quality; and average displacement of nesting material. The derivation of each measure from raw video footage is described below and provided in the Supplementary Methods.

### Wheel-derived DFI Measurements

Three parameters derived from analysis of wheel spins: average per-day distance run on the wheel; gait speed on the wheel; and circadian distribution of wheel-running activity. Wheel-running activity was monitored using an ML pipeline that identified the timing of individual rotations of the running wheel using a black stripe on the wheel’s surface (Figure 1A). In our video cages, the running wheel always appeared in the upper-right quadrant of the image, so all ML models in this pipeline were trained on and applied to only that quadrant of each video frame.

The wheel spin analytical pipeline combined semantic segmentation ML models using the “vgg_unet” architecture (32), an image classification ML model derived from mobilenet_v1 (33), and the parsing of a Hidden Markov Model using the Viterbi algorithm (34). The outputs of this pipeline were frame-by-frame annotations of whether a marker on the wheel was in the “top” or “bottom” position, which were used to infer rotations of the wheel. Pipeline details are provided in the Supplementary Text, along with specific performance evaluation.

Methods for calculation of the average per-day distance run on the wheel, gait speed on the wheel, and the circadian distribution of wheel-running activity are provided in the Supplementary Text.

### Floor-of-Cage Movement DFI Measurements

Two DFI parameters derived from analysis of movement across the floor of the cage: gait speed on the floor of the cage; and circadian distribution of movement on the floor of the cage. Tracking of the mouse as it moved about the cage provided the foundational data for multiple parameters. This tracking was achieved using an object-detection model based on ssd_mobilnet_v1 (33,35). More model details and methods for the calculation of gait speed on the cage floor are provided in the Supplementary Text.

Circadian movement on the floor of the cage was calculated using the same distance-traveled values described above for gait speed. The same method was applied as for the circadian distribution of wheel-running activity (see Supplementary Text), substituting distance-traveled for wheel-spins.

### Body Weight Change DFI Measurement

For the estimation of body weight and assessment of coat condition, pixels overlapping the mouse (ie, mouse masks; Figure 1B) were identified through a 2-step process, performed on each frame of video using the same object-detection model as for floor-of-cage tracking (33,35) and a semantic segmentation ML models using the “vgg_unet” architecture (32). For body weight, the masked area of the mouse was estimated on a per-frame basis using a pipeline that also included image classification models based on EfficientNet-B0 (36). Pipeline details are provided in the Supplementary Text, along with details of how the model was trained and evaluated.

For DFI, the rate of change of body weight was calculated across each designated DFI measurement period. Video frames were sampled once per minute, and estimates and accompanying weights were ascertained as described above for each video frame image. Rates of body-weight change were estimated using weighted linear regression with the time at which the video frame was recorded as the independent variable.

### Coat Condition and Nest Movement DFI Measurements

Coat condition was assessed in terms of image roughness, measured using Sobel gradients (37) as implemented by OpenCV (38), across the masked area of the mouse, determined as for the body weight pipeline described above. Pipeline details are provided in the Supplementary Text.

For the assessment of nest movement, a semantic segmentation model using the “vgg_unet” architecture (32) was trained to identify nest material (cotton in the video cages), and the movement of that material was measured through time. Pipeline details are provided in the Supplementary Text.

### Parameterization of Measurements Into Frailty Values

For each of the 8 components of DFI, the measured value was converted into a frailty score, meant to indicate observation of an exceptional value that reflects an abnormal, pathological state. Threshold values were determined ad hoc, with guidance provided by 2 age-stratified training sets (one of C57BL/6J mice, the other of J:DO mice; described above). To mimic the structure and concept of MFI and similar FIs, each of the 8 components was scored on a scale from zero to one, with zero indicating normal and one indicating frail behavior. In MFI, quantification of intermediate or ambiguous frailty was permitted, to a very limited extent, through the optional assignment of a score of 0.5. For DFI, threshold values were established for scores of either zero or one, and intermediate scores were linearly represented in terms of their relative proximity to those two thresholds. The overall DFI value was calculated as the average of the 8 parameterized scores. Those component scores and the overall score are provided for each measurement of DFI in Supplementary Table 4 for the C57BL/6J study and Supplementary Table 5 for the J:DO study.

Optimized DFI values were calculated as the mean of parameterized scores for the 5 best-performing components of DFI (see Main Text). Combined FI was calculated, for each pair of MFI and DFI values, as the mean of those two values (see details on pairing below).

Threshold values for each DFI parameter are described in the Supplementary Text. For each component of each DFI measurement, the preparameterized phenotype values are provided in Supplementary Table 6 for the C57BL/6J study and Supplementary Table 7 for the J:DO study.

### Data Properties and Statistical Analyses

For analysis of MFI versus age, all recorded and properly formatted MFI measurements were used, with age calculated as the difference between the animal’s cohorts’ date of birth and the date of the MFI assay.

For video monitoring, footage was streamed into cloud storage from a camera-enabled cage rack, with one camera per cage position. Data were organized by the device ID of the camera that collected it, and by date and time of acquisition. An electronic system maintained records of which mouse occupied which cage position at what times and those records were used to define DFI measurement period. For each DFI measurement, a mouse was recorded in a video cage for approximately 1 week, singly housed, following MFI measurement. For analysis of DFI, incomplete days of video at the beginning and end of the designated DFI measurement period were excluded from the analysis.

For the C57BL/6J study, video recording began immediately following the collection of MFI measurements (one per mouse). Three DFI measurements were taken from a single continuous period of video monitoring. Each period was 6 days (144 hours) long: the first began on the first full day of video recording, the second began at the end of the first, and the third began at the end of the second. These 3 measurements were separated from one another during data analysis, drawing from a continuous video stream across all 3 periods. One mouse died during the second observation period, so only one DFI value was recorded.

For the J:DO study, only DFI periods with at least 2 full days of observation were included; for observation periods with more than 6 full days of observation, only the first 6 full days were considered. Each DFI measurement was assigned to the first full day of video recording as the date of measurement. For analysis of DFI versus age, all DFI measurements meeting the criteria above were used, with age calculated as the difference between the animal’s cohorts’ date of birth and the first date of the DFI measurement date. For the J:DO study, a table of the measurement intervals that is compliant with the input requirements for the software system is provided in Supplementary Table 8.

For the comparison of MFI versus DFI in the J:DO study, measurement pairs were ascertained by seeking, for each DFI measurement, the MFI measurement from the same mouse with the closest date of acquisition. Only pairs of data collected fewer than 8 days apart were considered, with the DFI assay date corresponding to the beginning of DFI video acquisition. A table of qualifying MFI/DFI pairs is provided in Supplementary Table 9. For comparisons between age-normalized MFI and age-normalized DFI, linear regression was performed between age and the subset MFI or DFI data for which paired data had been found. The slopes calculated from those two regressions were used to calculate residuals of each datum versus its respective regression against age, and the MFI residuals were regressed against and correlated with the DFI residuals.

All regressions and correlations (“R” in the text refers to Pearson R) were calculated using the “scipy.stats.linregress” function from Scipy (39), which in addition to slope and intercept returns Pearson R and regression *p* value. Clustering of phenotypes based on a matrix of correlation coefficients was performed using Scipy’s “scipy.cluster.hierarchy” module: the “linkage” method was used with the “weighted” option, invoking hierarchical WPGMA clustering.

## Results

### DFI Values Correlated with MFI and Chronological Age in Male C57BL/6J Mice

Isogenic C57BL/6 is a commonly used mouse strain for FI studies (eg, (9–12)). Our first study evaluated the performance of DFI using male C57BL/6J mice, aged 148 days (*N* = 12), 508 days (*N* = 10), and ≥801 days (*N* = 9; mean age = 904 days, *SD* = 111 days). Each mouse was measured for MFI, then video recorded for 3 weeks continuously, allowing for the calculation of 3 independent DFI measurements per animal, taken from nonoverlapping 6-day windows. One mouse died during video recording, so only one DFI measurement was available for that mouse.

**Figure 2.**
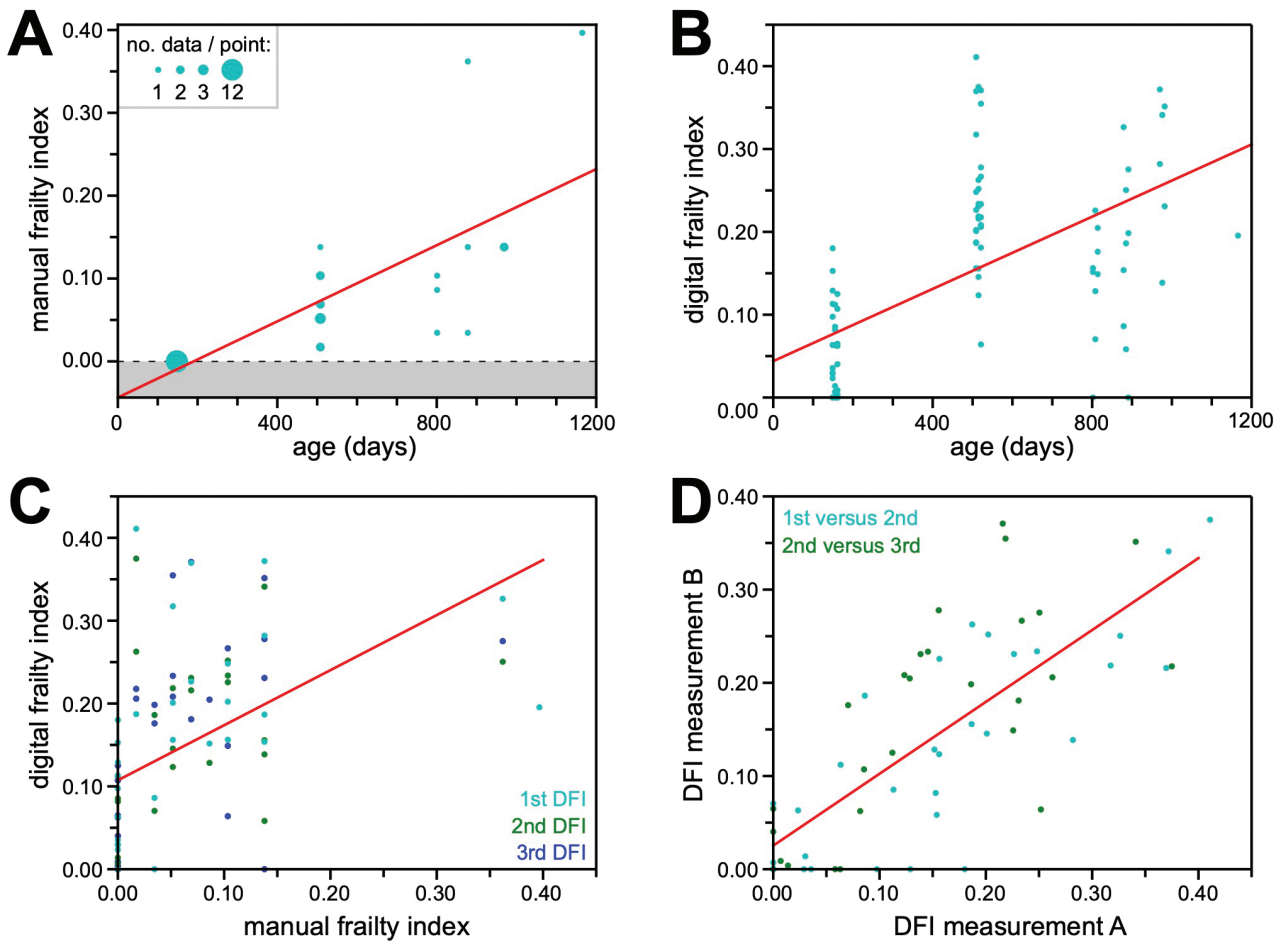
DFI positively correlated with MFI and chronological age in male C57BL/6J mice. (**A**) Chronological age (x-axis) versus MFI (y-axis). One measurement was taken per mouse in this experiment (see Method), and each dot represents one mouse/measurement. Redundant points are increased in size to indicate the number of data they represent, as indicated in the key. Regression line is in red (*R* = 0.76; *p* value = 8.8 × 10^−7^). (**B**) Chronological age (x-axis) versus DFI (y-axis). Up to 3 measurements were taken per mouse in this experiment (see Method), and each dot represents one measurement. Regression line is in red (*R* = 0.57; *p* value = 2.8 × 10^−9^). (**C**) MFI (x-axis) versus DFI (y-axis). In this experiment, one MFI measurement was taken per mouse, immediately followed by up to 3 consecutive DFI measurements (see Method). Each dot in this plot represents one DFI measurement, paired with the single MFI measurement from that mouse. Cyan dots are from the first DFI measurement, green dots are from the second, and blue dots are from the third. Regression line is in red (*R* = 0.48; *p* value = 1.3 × 10^−6^). (**D**) Correlation between consecutive DFI measurements. From an experiment in which 3 DFI measurements were taken per mouse from consecutive 6-day observation periods (see Method): points compare either the first and second measurements (cyan) or the second and third measurements (green). In both cases, the earlier measurement’s value is plotted on the x-axis and the later’s value on the y-axis. Regression line is in red (*R* = 0.77; *p* value = 8.2 × 10^−13^). DFI = digital frailty index; MFI = manual frailty index.

As has been generally reported for FIs including the clinical FI used here (10), our MFI measurements correlated positively with chronological age (Figure 2A; *R* = 0.76; *p* value = 8.8 × 10^−7^). Individual DFI measurements also correlated positively with chronological age (Figure 2B; *R* = 0.57; *p* value = 2.8 × 10^−9^). Individual DFI measurements from all 3 nonoverlapping observation windows also correlated positively with their paired MFI values (Figure 2C; *R* = 0.48; *p* value = 1.3 × 10^−6^).

To evaluate the within-animal consistency of DFI, we compared the values determined from consecutive, nonoverlapping, 6-day observation windows. This was under the assumption that animals’ frailty states would not change substantially over such short time intervals, but DFI would be required to assign values to those states based on independent sets of behavioral observations. The consistent behavior of DFI across all adjacent pairs of measurements (first-vs-second and second-vs-third) confirmed the consistency of this metric (Figure 2D; *R* = 0.77; *p* value = 8.2 × 10^−13^).

### DFI Values Correlated with MFI and Chronological Age in J:DO Mice of Both Sexes

**Figure 3.**
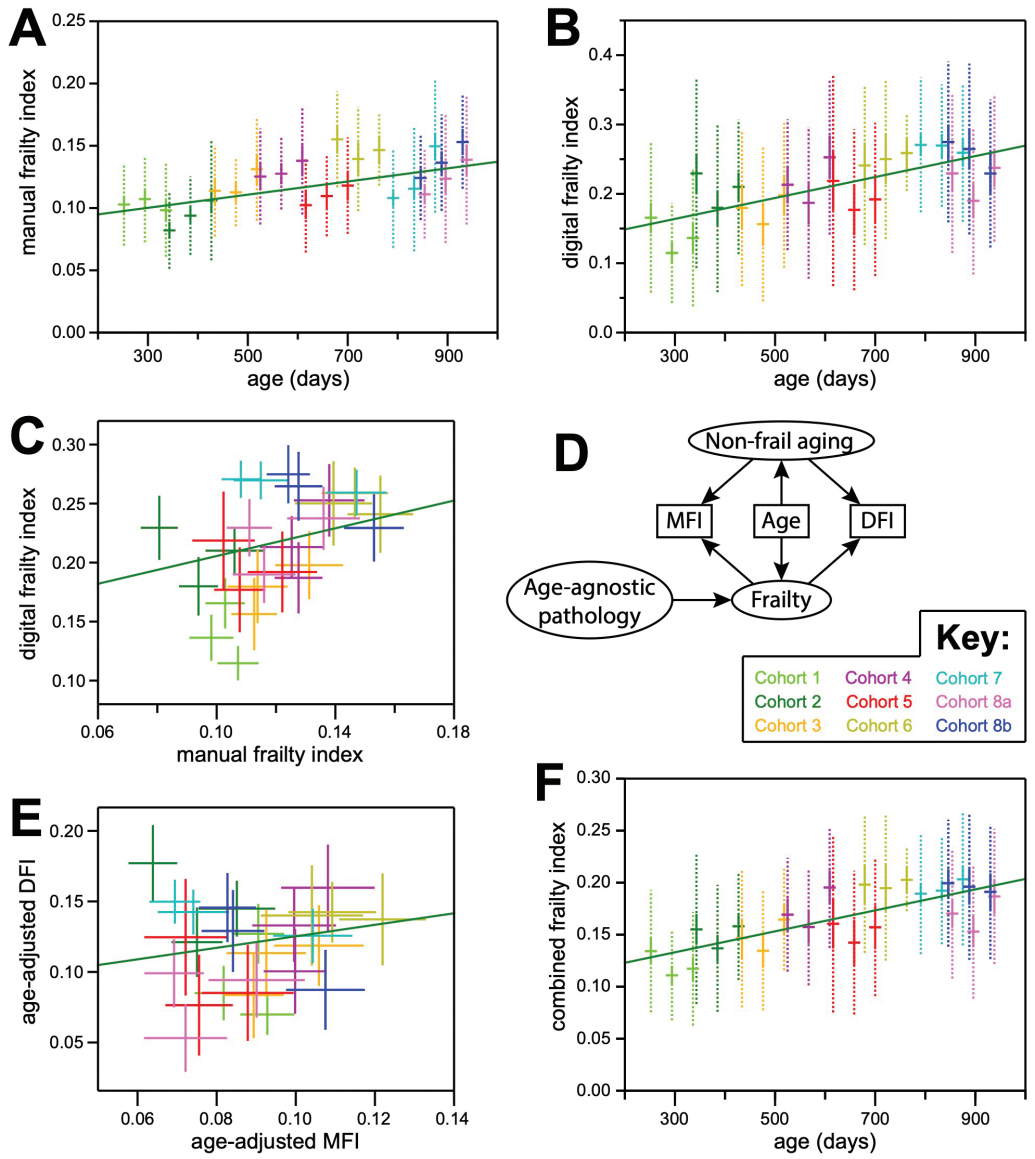
DFI positively correlated with MFI and chronological age in J:DO mice. (**A**) Chronological age (x-axis) versus MFI (y-axis). Mean (horizontal bars), standard errors (solid vertical bars), and standard deviations (dotted vertical bars) are shown for each of 3 measurement instances taken for each of 8 birth cohorts (with cohort 8 split into 2 subgroups: see Key). The linear regression of age versus MFI for all individual values is shown in green. (**B**) Chronological age (x-axis) versus DFI (y-axis). Plotted as in (A). (**C**) MFI (x-axis) versus DFI (y-axis). For each of 3 MFI/DFI measurements taken for each cohort, a cross is drawn whose center is the mean value on each axis for one cohort/measurement, and whose bars indicate the standard errors of those means along each axis. Colored according to the Key. The linear regression of MFI versus DFI for all individual values is shown in green. (**D**) A path diagram depicting a model for consideration of the possible relationships between MFI, DFI, and chronological age. See Results and Discussion. (**E**)Age-normalized MFI (x-axis) versus age-normalized DFI (y-axis), plotted as in (D). (**F**) Chronological age (x-axis) versus combined frailty index (CFI: the average of MFI and DFI; y-axis). Plotted as in (A). DFI = digital frailty index; MFI = manual frailty index.

For our second of two studies, in order to evaluate the robustness of DFI across diverse mice, data was collected from J:DO mice, including 587 measurements of MFI, taken across 213 mice, aged from 252 to 938 days. Though shallower than with C57BL/6J, the MFI values for J:DO mice correlated positively with chronological age (Figure 3A; *R* = 0.27; *p* value = 1.7 × 10^−11^). This correlation held for both females (348 measurements from 128 animals; *R* = 0.37; *p* value = 3.8 × 10^−13^) and males (239 measurements from 85 animals; *R* = 0.23; *p* value = 2.7 × 10^−4^).

Our J:DO study also included 576 instances of measured DFI in J:DO mice. Similar to MFI, DFI correlated positively with chronological age (Figure 3B; *R* = 0.29; *p* value = 8.0 × 10^−13^). Again, this relationship held for both females (341 measurements from 136 animals; *R* = 0.30; *p* value = 1.4 × 10^−8^) and males (235 measurements from 88 animals; *R* = 0.26; *p* value = 4.6 × 10^−5^).

As with our male C57BL/6J experiment, this D.O. experiment included repeated DFI measurements on each animal, allowing for technical reproducibility to be evaluated. In contrast to the C57BL/6J experiment, in which subsequent observations were taken immediately, the D.O. study included 6-week intervals in between MFI and DFI measurements. These longer gaps provided greater opportunity for the true frailty state of an animal to change in between observations. Nonetheless, significant correlation was observed for DFI between the first and second measurements (*N* = 186; *R* = 0.53; *p* value = 5.7 × 10^−15^); also between the second and third measurements (*N* = 169; *R* = 0.52; *p* value = 5.2 × 10^−13^). Consecutive MFI measurements similarly correlated, between the first and second measurements (*N* = 186; *R* = 0.55; *p* value = 7.5 × 10^−16^); also between the second and third measurements (*N* = 169; *R* = 0.62; *p* value = 4.6 × 10^−19^).

Our study included 567 instances for which a DFI measurement was taken within 7 days of an MFI measurement of the same mouse (Supplementary Table 9). DFI values were positively correlated with MFI values (Figure 3C; *R* = 0.22; *p* value = 1.8 × 10^−7^). This correlation held for both females (337 MFI/DFI pairs from 126 animals; *R* = 0.29; *p* value = 8.2 × 10^−8^) and males (230 MFI/DFI pairs from 84 animals; *R* = 0.19; *p* value = 3.1 × 10^−3^).

The correlation of both MFI and DFI with chronological age (Figure 3C) implied that the correlation between MFI and DFI could have been mediated by chronological age rather than the intended mediator, physical frailty (Figure 3D). To evaluate that possibility, the residuals of both MFI and DFI from the regression of each with chronological age were compared. Though weakened, the correlation between these residuals remained positive and statistically significant (Figure 3E; *R* = 0.15; *p* value = 2.7 × 10^−4^).

Significant correlations between DFI and the benchmarks (age and MFI) were reassuring, but modest *R* statistics suggested room for improvement. To assess the collective potential for improvement of either/both metrics, we averaged their outputs into a combined FI and evaluated it versus chronological age. The extent of correlation was greater (*R* = 0.34) and more significant (*p* value = 3.5 × 10^−17^) than for either individual score (Figure 3F), suggesting opportunities for further improvement in this field.

### Individual DFI Components Provided Variable Utility in the Study Cohorts

**Figure 4.**
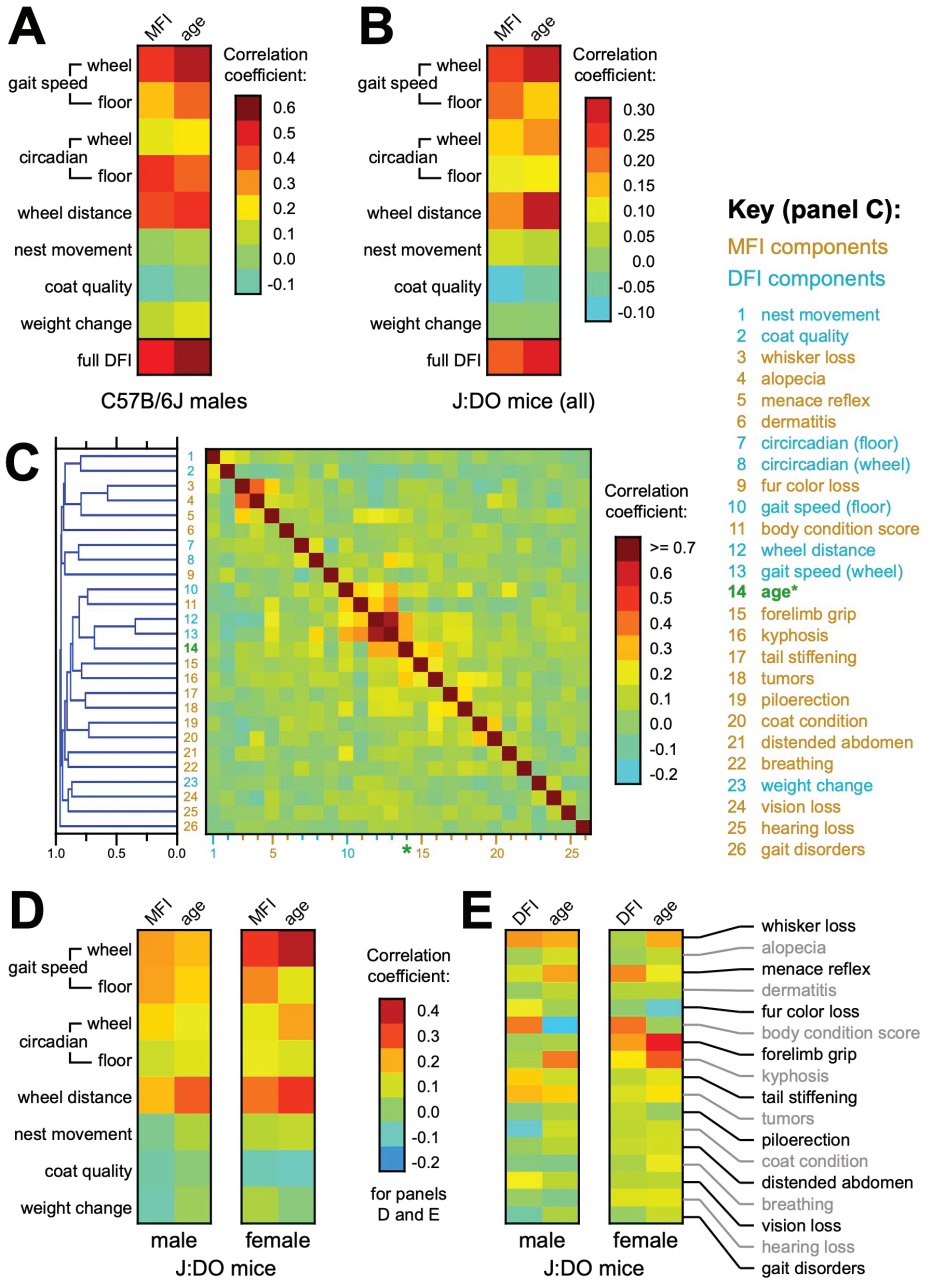
Most DFI components positively correlated with MFI and chronological age. (**A**) A heat map indicating the correlation coefficients between individual components of the DFI (and, at bottom, the full DFI) versus MFI and chronological age, among C57BL/6J male mice. (**B**) A heat map indicating the correlation coefficients between individual components of the DFI (and, at bottom, the full DFI) versus MFI and chronological age, among J:DO mice of both sexes. (**C**) Hierarchical clustering (left) of individual MFI and DFI components, and chronological age, based on the correlation coefficients depicted in the heatmap (right). MFI components are labeled in orange; DFI components are labeled in blue; chronological age is labeled in green. Only MFI components observed with non-zero values >5 times across the study are included. (**D**) Heat maps indicating the correlation coefficients between individual components of the DFI versus MFI and chronological age, among J:DO mice, independently analyzed for each sex. (**E**) Heat maps indicating the correlation coefficients between individual components of the MFI (those included in panel C) versus DFI and chronological age, among J:DO mice, independently analyzed for each sex. DFI = digital frailty index; MFI = manual frailty index.

Our implementation of DFI included 8 measurements, reflecting 4 arenas of function: physical fitness capability, environmental engagement, circadian rhythm, and body condition (2 measurements per arena; see Method). In all cases, methods were developed and parameterized using separately-collected training data from both C57BL/6J and J:DO mice, then evaluated in a small population of C56BL/6J male mice and a large population of J:DO mice of both sexes. The relevances of individual parameters to chronological age and MFI varied considerably when analyzed using data from C57BL/6J males (Figure 4A). Generally, the gait/wheel and circadian components of the DFI were highly correlated with both MFI and chronological age; while components focused on the movement of nesting material, coat quality, and change in body weight change shared little correlation with those benchmarks. Amongst J:DO mice, those correlations between DFI parameters and age/MFI benchmarks were diminished in scale versus their C56B/6J counterparts (Figure 4B). Nonetheless, the same pattern of high correlation for gait/wheel/circadian components and low correlation for other components was shared between C57BL/6J males and J:DO mice (Figure 4A and B). We discuss the performance of each DFI parameter in greater detail in the Supplementary Text.

Using data from the J:DO dataset, the clustering of DFI and MFI components based on the all-by-all correlation matrix revealed little structure (Figure 4C). Few instances of substantial correlation between frailty traits were observed. Versus either the higher-order parameters (MFI, chronological age) or the component parameters of MFI with seeming relevance, multiple explanations may account for missing correlation aside from true irrelevance of the traits. Among them, poor model performance at measuring phenotypes; inappropriate parameterization of frailty thresholds; or sparsity of instances of certain types of frailty in this cohort, in spite of its size.

Like the comparison of individual parameters’ performances between C57BL/6J and J:DO mice (Figure 4A and B), the comparison of performance profiles between male versus female J:DO mice revealed similar trends (Figure 4D). Though less-powered, a similar comparison of the predictive power for individual MFI parameters, evaluated against the benchmarks of DFI and age, revealed similar patterns between the sexes (Figure 4E).

## Discussion

Here, we implemented a DFI for mice based entirely on the computational analysis of continuously collected home-cage video footage. The performance of DFI was evaluated against both chronological age and MFI and found to correlate with both metrics in a cross-sectional study using male C57BL/6J mice, with age effects similar to what has been reported for this strain (eg, (10)). A large cross-sectional study using male and female J:DO mice validated significant correlations of DFI with the benchmarks, albeit with lesser coefficients. Success with genetically diverse mice, with varying builds and coat colors, suggested that DFI will be applicable to a wide range of mouse models.

FIs are so named because they are meant to reflect a lack of physical well-being (2,9). They are not explicitly designed to be predictors of chronological age, but the physiological decline that accompanies age results in a natural and expected age dependence (eg, (1,4,8–10)). However, it has been shown that even the components of a traditional FI can be weighted to optimize age prediction (40). Allaying the concern that age mediated the correlation between MFI and DFI, correlation between them was maintained after each was normalized to age (Figure 3E).

Home-cage DFI represents a practical step forward for longitudinal health assessments in terms of vivarium operational efficiency and scalability. Since observations are taken in a home-cage environment, highly trained research personnel are not required, just husbandry staff for feeding and care. Furthermore, in order to be meaningful, traditional FI requires consistent scoring across large and longitudinal cohorts. The practices that ensure high reproducibility among raters (good communication, similar educational background, etc.) (13–15) are more difficult to maintain as experiment size, and therefore team size, increases. In contrast, DFI can be uniformly applied regardless of the experimental scale. This advantage applies doubly to longevity/healthspan studies, as it can be difficult to guarantee consistent staffing across multi-year mouse life spans.

### Consistencies and Differences Across Strains and Sexes

DFI effectively detected age-related frailty increases in both a traditional model for mouse FI studies, C57BL/6J, as well as in genetically diverse J:DO mice. For C57BL/6J, the strong correlation with age established by the creators of the clinical FI used for MFI (10) was reproduced in both our MFI and DFI data. The pattern observed with age for DFI in our C57BL/6J study was a sharp increase between ~180 days and ~500 days of age, with very little increase after that, beyond 800 days of age (Figure 2B). In the reference data provided by Whitehead et al. (10), for which data were collected at very similar ages (Figure 1 of (10)), that pattern is better matched by the 8-item performance-based FI developed by Parks et al. (9) (Figure 2A of (10)) than for the more gradual, continuous increase in clinical FI (Figure 3A of (10)). The relationship between MFI and DFI (Figure 2C) is strikingly similar to the plot of clinical versus performance-based FIs from Whitehead et al. (Figure 4A of (10)). This was not surprising, as DFI is also a reduced-feature-set version of FI that focuses on performance.

Common patterns of performance across the individual DFI parameters (Figure 4A and B) suggested that shared physiological deficits accompanied age in both strains—and, with one of those strains being J:DO, that those deficits were relevant across a wide swathe of genetic backgrounds. Additionally, those patterns were broadly shared between male and female J:DO mice. Notably, the biggest differences between the sexes were enhanced signals in females for wheel performance (speed and total distance; Figure 4D) in DFI, and an enhanced signal for “forelimb grip” in MFI (Figure 4E). These enhanced signals seemed most relevant to peak muscle function, which is also a noted harbinger of female frailty in humans (41).

Despite the common patterns with C57BL/6J males, the overall magnitude of correlations linking DFI and its components to age and MFI were diminished in the J:DO background. This was not due to insufficient performance by DFI: versus performance in C57BL/6J mice, the correlation with age was equally dampened in J:DO for both MFI and DFI. Genetic variance influences physiological health and its age-related decline in J:DO mice (42). It would not be surprising for that extra variance to contribute noise and therefore decrease the magnitude of valid physiological correlations.

### Limitations of Current DFI: Data Acquisition, Single-Animal Housing, and Parameter Scope

Like many limited-application ML models, those at the foundation of DFI rely on a consistent visual environment to work effectively, without further training. Our models were all trained on data acquired using the Vium system, and they would not be expected to work on footage collected from cages that did not look similar. The availability of compatible video systems may limit the impact of our DFI implementation.

Home-cage DFI is noteworthy for its ability to almost eliminate animal handling-related stress as an experimental confounder (21–28). The recent development of an entirely open-field-based FI technique (20) also greatly minimizes this source of stress, but still requires handling immediately prior to the assay, and also introduces the stress of a novel, brightly lit environment (29). However, due to the time interval required to collect effective DFI measurements (~a week), DFI introduced a potentially new source of stress: single housing. Mice are social animals, and single housing can have a wide range of negative psychological and physiological consequences (43–45).

Some studies on the effects of short-term single housing (~1–2 weeks) indicate little additional stress from this environment (46–48), in contrast to the substantial stress associated with long-term single housing (≥8 weeks) (44,45). As illustrated by our J:DO experiment, intermittent short-term single housing is a compatible experimental design with current DFI. Also, the stresses of single housing must be balanced against the stresses and dangers of group housing, especially for male mice, due to aggressive behavior: the effects of social stresses and best practices can be strain-specific (43,49). For the male J:DO mice used in our experiment, best practice is single housing in the home cage (eg, (50,51)) due to documented aggressive behavior (52). Group housing may still be practiced if littermates are co-housed from an early age (53), but animals with such a history are not always available. These considerations aside, it would be undoubtedly preferred for DFI to be amenable to group housing. That remains an important future goal for this system.

The current selection of parameters measured by DFI represents a second domain for future improvement. Of the 8 DFI parameters, 3 provided little statistical signal: nest movement, coat quality, and body weight change (Figure 4; Supplementary Figure S4). Connections to age-related decline exist for all 3 of these parameters in the literature, and similar metrics are often included in FIs (eg, (10,11,54,55)). Multiple factors may have contributed to these shortcomings, including inappropriate timing to optimally observe relevant activity; sparsity of adverse events across any of our data sets; and inadequacy of our implementations. The latter represents an opportunity for future improvement; see Supplementary Results for more detailed discussion.

Traditional FIs also suffer from uneven performance across their parameters (10,40). However, the plethora of parameters can statistically compensate for under-performance by individuals. Only 8 parameters in DFI, while not unprecedented (9), were fewer than would be found in a typical FI (eg, (10,11)), implying that expansion of the parameter set as a target for future improvement. Supporting this notion was the improved correlation with chronological age when DFI and MFI were combined (Figure 3F).

Two potential novel parameters, that are visually apparent and effective as frailty indicators, would be kyphosis and tail-stiffening (40). Grooming behavior is a third: although coat condition was not effective in our initial implementation, ML methods that directly monitor grooming activity can add sensitivity (56). A nest-focused parameter based on quality scoring rather than movement could also be more effective (57). Finally, grimace could be added. Rare frames with good views of the mouse’s face would first need to be auto-detected, but grimace effectively detects mouse frailty (16) and has a history of success with ML-based classification (17).

There are many potential ways to improve DFI, enumerated above. Nonetheless, in its current state, it achieves the performance requirements to justify future application. It also serves as a proof of principle for the aspirational concept of a digital vivarium (22), providing a refined, scalable and automated platform to study the biology of aging.

## Supplementary Material

glad035_suppl_Supplementary_Materials

## Data Availability

Code and accompanying ML models for running DFI analysis on a set of videos is available at https://github.com/graham-calico/DFI_v1.
