# Supplementary material for: An Automated, Home-Cage, Video Monitoring-based Mouse Frailty Index Detects Age-associated Morbidity in C57BL/6 and Diversity Outbred Mice
Source: J Gerontol A Biol Sci Med Sci. 2023 Jan 27;78(5):762–70. doi: 10.1093/gerona/glad035 (PMC10172975; doi:10.1093/gerona/glad035)

Supplemental Text and Figures for:

An automated, home-cage, video monitoring-based mouse frailty index detects age-associated morbidity in C57BL and Diversity Outbred mice.

J. Graham Ruby, PhD*, Andrea Di Francesco, PhD, Paulo Ylagan, BS, Angela Luo BA, Robert Keyser, BS, Owen Williams, BA, Sarah Spock, BS, Wenzhou Li, PhD, Nalien Vongtharangsy, BS, Sandip Chatterjee, PhD, Cricket A. Sloan, MS, MS, Charles Ledogar, MEng, Veronica Kuiper, BS, Janessa Kite, MSc, Marcelo Cosino, Paulyn Cha, BS, Eleanor M. Karlsson, DVM*

Calico Life Sciences LLC, South San Francisco, California 94080,

*to whom correspondence should be addressed: graham@calicolabs.com and ellie@calicolabs.com

Supplemental Methods 2

Mice, animal husbandry, and in vivo study design: housing details 2

MFI score details 2

Calculation of Digital Frailty Index (DFI) score 3

Wheel-derived DFI measurements: pipeline details 3

Floor-of-cage movement DFI measurements: model & method details 4

Body weight change DFI measurement: pipeline and evaluation details 5

Coat condition: pipeline details 6

Nest movement: pipeline details 7

Parameterization of measurements into frailty values: threshold details 7

Supplemental Results & Discussion 8

Individual parameters performance 8

Walking and running statistics robustly captured age-related decline. 8

Circadian regulation of movement decreased with age. 8

Frailty measurements of coat quality, body-weight change, and nest movement showed no statistical change with age. 9

Performance improves with removal of low-performing parameters 10

References 11

Supplemental Figure Legends 12

Supplemental Figures 14

Supplemental Methods

Mice, animal husbandry, and in vivo study design: housing details

All mice were housed in solid-bottom 100% PET plastic, BPA-Free IVC cages (Innovive) . All cages and bedding were irradiated prior to use. Mice were housed on 1/8-in. corn cob bedding (Innovive, San Diego, CA), received acidified (pH 2.5 to 3.0) reverse osmosis–purified water from water bottles (Innovive, San Diego, CA), and were fed irradiated diet chow (LabDiet Pico 5L0D, Purina, St. Louis, MO). Mice received 8g shredded paper nesting material and a paper hut enrichment (Enviro-Dri and Shephard Shack, Shephard Specialty Papers, Watertown, TN) while in their non-video cages. Mice were provided two cotton nesting squares (NES 3600, Ancare, Bellmore, NY) for enrichment, along with a climbing ladder and running wheel (Vium) while in video cages. All mice were housed under a 12:12-h light:dark cycle at a density of 1 to 4 mice per cage in a temperature-controlled vivarium, in compliance with the Guide for the Care and Use of Laboratory Animals. Male mice were singly housed throughout the study, while female mice were single housed in video cages (1 week out of every 6 weeks for a total of 18 weeks). Animal cages were changed every 2 weeks within a cage change station (NuAire, Plymouth, MN). Mice were transferred between cages using red transparent acrylic tunnels (Bio-Serv, NJ) or by cupping technique.

MFI score details

For the C57B/6J and J:DO studies, manual frailty was assessed as described in the original 31-item mouse clinical FI from Whitehead et al, omitting two parameters that rely on population-specific statistics (body weight and temperature). Mice were allowed to acclimatize to the testing room for 30-45 minutes before testing. Observations were carried out on an open bench at the same time of the day, between 9-11am. Mice were scored 0, 0.5, or 1 based on the severity of deficit they showed in each of the 29 items, with 0 representing no sign of deficit, 0.5 mild deficit and 1 severe deficit. Those 29 items were: alopecia, loss of fur color, dermatitis, loss of whiskers, coat condition, breathing rate/depth, mouse grimace scale, piloerection, tumors, distended abdomen, kyphosis, gait disorders, tremor, vestibular disturbance, tail stiffening, cataracts, corneal opacity, eye discharge/swelling, microphthalmia, nasal discharge, malocclusions, rectal prolapse, vaginal/uterine/penile prolapse, diarrhea, body condition score, forelimb grip strength, menace reflex, vision loss, and hearing loss.

Body weight was measured at the beginning of each MFI test, as was body surface temperature, averaging three readings obtained with an infrared temperature probe directed at the abdomen. Because of the potential for genetic variance in the J:DO population to confound the interpretation of specific values for these parameters as frailty-related, they were not included in the MFI scores but are provided in Supplemental Tables 1(C57B/6J) and 3 (J:DO), along with other MFI scores.

Calculation of Digital Frailty Index (DFI) score

Wheel-derived DFI measurements: pipeline details

The pipeline first applied two independent, two-channel image-segmentation models: the first to draw a mask covering the open face of the running wheel, the second to draw a mask covering the black stripe. Those masks were used to construct a synthetic image, with the wheel-face mask displayed in the blue channel and the black-stripe mask displayed in the green channel. Overlap of the two masks, which occurred when either the stripe was under but still visible through the translucent-plastic wheel or when a black mouse was confused as a stripe, therefore appeared as cyan in the synthetic image. That synthetic image was provided as input to an image-classification ML model, which returned probabilities for each of two classes, corresponding to the black stripe being on the “top” or “bottom” of the wheel. Those probabilities were used as emission probabilities for those two respective states, and a two-state Hidden Markov Model (HMM) was parsed across those emissions using the Viterbi algorithm (Viterbi, 1967) across all frames of each full 10-minute video. From the resulting parse, every full transition cycle (from “top” to “bottom” and back to “top”, or vice-versa, depending on the initial state for that video) was recorded as one spin of the wheel, across a length of time determined by the video frame rate and the number of frames traversed through the spin.

The two image-segmentation models described above, for the wheel’s face and marker, were each trained using the tool provided by the “image-segmentation-keras” code base (https://github.com/divamgupta/image-segmentation-keras commit f04852d from September 6, 2019), specifying the “vgg_unet” model architecture (Simonyan, 2014), with input widths and heights of 128 and 96, respectively. These models were trained on and applied to only the upper-right quadrant of the images, which consistently contained the entire wheel. The classification model described above was a re-trained version of the TF-Slim implementation of mobilenet_v1 with a depth multiplier of 1.0 and an input size of 224 by 224 (Howard et al, 2017). The wheel-position HMM had initial state probabilities of 0.325 for “top” and 0.675 for “bottom”. Transition probabilities were set to ⅔ when returning to the same state and ⅓ when changing state.

The wheel-spin ML pipeline was trained on frames taken from video of C57BL/6J mice. It was tested on both an independent set of C57BL/6J mice and video from three cohorts of differently-aged J:DO mice, using manually spin counts for 100 10-second clips from each cohort. Correlation coefficients (Pearson’s R) between the model- and manual-derived spin counts were 0.994 for the C57BL/6J cohort and 0.995 (young), 0.986 (middle), and 0.980 (old) across the three J:DO cohorts (Supp. Fig. S1B). Across all four test cohorts, accuracy increased with the mean number of manually-counted spins per clip – 5.73 spins/clip for the C57BL/6J cohort and 1.52 spins/clip (old), 3.15 spins/clip (middle), and 6.08 spins/clip (young) for the J:DO cohorts (Supp. Fig. S1C). By inspection: most errors were single frames at the beginning or end of the clip for which the status of transition to a new state (“top” or “bottom”) was ambiguous.

To calculate the average per-day distance run on the wheel: for each ten-minute video, frame-by-frame parsed HMM states were analyzed to count wheel spins by counting the number of instances of the state changing (between the marker being on the “top” or “bottom” of the wheel) and dividing by two. The wheel-spin sums from all videos within each designated DFI measurement period (approx. one week) were summed and divided by the total length of footage from that DFI measurement period, in days.

To calculate gait speed on the wheel: for each ten-minute video, frame-by-frame parsed HMM states were analyzed to identify the number of frames that elapsed for each complete spin (number of frames divided by frames-per-second for the video). For all complete spins observed across the designated DFI measurement period, the median length of time t_{sp_med} was used to define the typical gait speed (1 / t_{sp_med}).

To calculate the circadian distribution of wheel-running activity: distance in wheel spins was determined for each 10-minute video, as was done for total distance. For each designated DFI measurement period, day versus night periods were defined as alternating non-overlapping, 12-hour blocks of clock time, and their phase was allowed to vary by 10-minute increments across the full 24-hour cycle. For each phase, the average activities across “day” and “night” videos were calculated, and the differences between the two were recorded. The maximum absolute value from those differences was used to define circadian activity, normalized to twice the weighted average across all “day” and “night” values.

Floor-of-cage movement DFI measurements: model & method details

Tracking of the mouse as it moved about the cage was achieved using an object-detection model, trained by transfer learning using the COCO-trained ssd_mobilnet_v1 model (downloaded from http://download.tensorflow.org/models/object_detection/ssd_mobilenet_v1_coco_11_06_2017.tar.gz) (Liu et al, 2016; Howard et al, 2017) using images taken from video feeds. Since all video footage was taken of singly-housed mice for this study, only the most confidently predicted box was used from each frame, and it was used regardless of its confidence score.

To calculate gait speed on the cage floor, the position of the mouse was determined for each frame of video using the center point of the highest-confidence box drawn by the mouse-detector model. For each frame in a 10-minute video, a refined position estimate was calculated using the mean position of the mouse across 15 adjacent frames (this truncated seven frames each from the beginning and end of each video). Using those refined positions, movement between each pair of frames was calculated as the linear distance between the positions in the two frames, in units of pixel length, using the Pythagorean theorem. Per-second distances were calculated for each non-overlapping second of video, using the integer of the video-encoded frame rate (fps), multiplied by the ratio of the float-encoded fps to its integer value (to compensate for under-estimates of distance due to frame-unit truncation). Gait speed was calculated as the weighted average of distance-per-second values, weighted by the distance-per-second value, which was mathematically equivalent to calculating the average gait

speed per unit of distance covered. These weighted averages were taken across all per-second estimates available across a day. Overall gait-speed estimates for a mouse across a frailty-measuring time segment were calculated as the weighted averages of all per-day estimates from the time interval, weighted to the total distance traveled per day.

Body weight change DFI measurement: pipeline and evaluation details

For the estimation of body weight and assessment of coat condition, pixels overlapping the mouse (i.e. mouse masks) were identified through a two-step process, performed on each frame of video. First, the most confidently predicted bounding-box from the mouse-detector model described above was used to isolate a subset of the frame image, corresponding to the area of the bounding box extended by 20% along each dimension (10% extension in each direction). Second, that sub-image, which constituted a zoom-in on the mouse, was fed as input to an image segmentation model that would provide a mask of the mouse as output. That segmentation model was trained using "image-segmentation-keras" as described above, again specifying the "vgg_unet" model architecture (Simonyan, 2014), with input width and height both set to 256. That model was trained & tested using similarly-boxed images of J:DO mice and achieved a mean IoU of 0.905 (SD 0.050) across 296 test images.

For the per-frame body weight estimate, the area of the mouse mask was normalized to remove fish-eye lens effects, based on each pixel's position in the original image. The area of each pixel *a* was up-weighted using the following equations, where x_{eq} and y_{eq} are the x and y coordinates of the pixel in the 864 pixel (width) by 648 pixel (height) image, relative to the center of the image, and the focal length variable *f* was assigned a value of 415.0:

$$r_{eq} = \sqrt{y_{eq}^{2} + x_{eq}^{2}}$$
$$r_{rec} = f * tan(r_{eq} / f)$$
$$a = (r_{rec} / r_{eq})^{2}$$

That measurement of area of the mouse in the image, in pixels, was then converted to voxels by accounting for the ratio of cross-sectional area to volume for a sphere ($\pi * r^2$ versus $4/3 * \pi * r^3$), by raising the pixel area to the power of 1.5 (the constant 4/3 was ignored). Based on regression with training data (description of training data set to follow), the resulting volume in voxels was converted to body weight in grams using the conversion factor: $1.309 * 10^{-5}$ voxels per gram.

For each body weight estimate, an image classification model was applied to identify and compensate for the largest-error estimates. This model was trained empirically by applying the above model to the training data set and categorizing images based on the magnitude and direction of the estimates' errors. Three classes were defined, separating training-set images based on errors >1.8-fold from the true value (too low for class "a"; within that range for class "b"; too high for class "c"). A classification model was trained using transfer learning, starting from EfficientNet-B0 (Tan & Le, 2019; downloaded from

https://tfhub.dev/google/efficientnet/b0/classification/1). Input images for that model were modified versions of the full original image, with the green and red channels of each pixel set to its grayscale value (average brightness value across the three color channels) and the blue value set to maximum brightness (255) in masked pixels and minimum brightness (0) in un-masked pixels (e.g. Main Fig. 1D). That modified image was classified as likely to be either an over-estimate, under-estimate, or appropriate estimate of body weight. Body weights from over-estimate images were multiplied by 0.4, and body weights from under-estimate images were multiplied by 2.174, based on training data error averages in these categories.

The same modified image was used by a second classification model to assign a reliability weight to the body weight estimate. Two classes were defined: “mouse” or “other”. Training and input images were colored in the same manner as input images for the estimate-error classification model above, with the segmented “mouse” pixels differentially colored. Training images were manually sorted based on a human rater’s opinion of whether the mask substantially covered the mouse and not substantially other parts of the image. The classification model was again trained using transfer learning, starting from EfficientNet-B0 (Tan & Le, 2019). In the DFI-scoring pipeline, images classified as “other” were assigned reliability weights of zero. Images classified as “mouse” were assigned a reliability weight w_{rel} derived from the confidence score c assigned to that class by the model: $w_{rel} = 2*(c - 0.5)$. Reliability weights were recorded along with their corresponding body weight estimates for use in weighted regression or mean estimates, performed later.

Body-weight prediction models were trained and evaluated using an independent data set of J:DO mouse videos that were collected within a day of a manually-ascertained body weight measurement. From a pool of 2,246 weight measurements with matched video, 14,321 frames corresponding to 974 ground-truth weights (37.6g mean; 8.6g SD; 19.18g minimum, 71.24 maximum weights) were selected for training, with frames corresponding to the same weight measurement spaced at least 1.5 hours apart. For testing, 230 weight measurements were selected that did not overlap with the training set, each of which had at least 50 ten-minute video files captured on the same date as the weight measurement. For evaluation, body weights were estimated from the first frame of each ten-minute video on the calendar day (up to 144 predictions), and the weighted mean of the predictions from each mouse & day was compared to the ground-truth measurement for that mouse on that day.

Coat condition: pipeline details

For the assessment of coat condition, the mouse detector model used above in the first step of mouse identification was again used to define a zoomed-in image of the mouse (as above: extended by 20% along each dimension). Sobel gradients (Sobel & Feldman, 1968) at each pixel were calculated using the OpenCV “Sobel” function (Bradski, 2000), along the x-axis (s_x) and y-axis (s_y) separately, then calculating the pixel value s_p as $\sqrt{(s_x^2 + s_y^2)}$. The overall Sobel value s_m was calculated as the mean of s_p values for all pixels overlapping the mouse mask, defined as described for body weight. The brightness of the mouse’s coat c_{br} was also calculated as the median pixel intensity for all pixels overlapping the mouse mask. For both s_m

and c_{br}, the overall value for an animal, for a designated DFI measurement period, was taken as the mean of all estimates collected from across that period, with one frame sampled per minute. Due to systematic bias of the intensity of Sobel gradients across mice of different coat colors (Supp. Fig. 1D), the final s_m value was adjusted based on coat color, as ascertained by coat brightness. For mice with an average c_{br} less than 30 (black mice), the s_m value was increased by 2; and for mice with an average c_{br} greater than 70 (albino mice), the s_m value was increased by 4. For the remaining mice (agouti), the s_m value was not adjusted.

Nest movement: pipeline details

For the assessment of nest movement, a semantic segmentation model was trained to identify nest material (cotton in the video cages). Nest material was manually labeled for training and test-set images from an independent video data set. The segmentation model was trained using “image-segmentation-keras” as described above, again specifying the “vgg_unet” model architecture (Simonyan, 2014), with input width set to 768 and input height set to 512. That model achieved mean IoU’s of approximately 0.75 across two separately-annotated test sets (both manually annotated, months apart but by the same rater). The position of nest material was taken for each frame as the mean position of masked pixels. Positions were estimated for each ten-minute video from across each designated DFI measurement period: for each video, the position was calculated as the average of positions from frames sampled once per minute across the video. Position was specified in terms of the pixel grid of the source images. Displacement was measured between time-adjacent videos, in pixels per ten-minute interval. In cases of a missing video file, displacement rate was calculated between nearest-time-adjacent videos, with the displacement rate still calculated per ten-minute interval. The output value was the average of all displacement-rate values obtained across the designated DFI measurement period.

Parameterization of measurements into frailty values: threshold details

For gait speed on the wheel, frailty values of zero and one were thresholded at 0.5 and 0.1 spins per second, respectively. For gait speed on the floor of the cage, frailty values of zero and one were thresholded at 60 and 20 pixel-lengths per second, respectively. For circadian behavior on both the wheel and the floor of the cage, frailty values of zero and one were thresholded at circadian ratios of 0.6 and 0.2, respectively. For total wheel-running distance, frailty values of zero and one were thresholded at 5,000 and zero spins per day, respectively. For nest movement, frailty values of zero and one were thresholded at 7.5 and 2.5 pixel-lengths per ten-minute interval, respectively. For coat quality, frailty values of zero and one were thresholded at Sobel values of 20 and 25, respectively. For body weight change, frailty values of zero and one were thresholded for the absolute value of the per-day weight change, at 1 and 2 grams per day, respectively. For each component of each DFI measurement, the pre-parameterized phenotype values are provided in Supplemental Tables 6 (C57B/6J) and 7 (J:DO).

Supplemental Results & Discussion

Individual parameters performance

Walking and running statistics robustly captured age-related decline.

Voluntary wheel-running is an activity known to be influenced by the physical health of mice (Greenwood & Fleshner, 2019). Our capture of elapsed time for each spin of the wheel (see Methods) permitted multiple facets of health and behavior to be probed using those data. First, the total amount of wheel running could be determined, measured as the total number of spins per day. This parameter captured an aspect of environmental engagement (quantity of interaction with an enrichment item) while also presumably reflecting physical endurance and athletic performance. In both our C57BL/6J and J:DO training sets, this feature declined sharply with chronological age, appearing to approach zero asymptotically as mice became old (Supp. Fig. S2A). When parameterized to frailty, strong correlation with chronological age was maintained (Supp. Fig. S2B; R = 0.32, p-value = $1.4 * 10^{-14}$), and correlation with MFI was achieved (R = 0.18, p = $1.8 * 10^{-5}$).

Gait speed on the wheel, interpreted in terms of athletic performance, was also derived from the wheel rotation frequencies (see Methods). Like total wheel-running, this parameter declined sharply with chronological age in both C57BL/6J and J:DO training-set mice (Supp. Fig. S2C). This relationship was maintained when parameterized to frailty (Supp. Fig. S2D; R = 0.32; p-value = $8.9 * 10^{-15}$), and correlation was achieved with MFI (R = 0.24; p-value = $3.3 * 10^{-9}$). Gait speed on the wheel has a lower-intensity counterpart in ambulation around the floor of the cage. The context is different (slower ambulation in the context of everyday activity, versus purposeful exercise-based enrichment), but the relationships with chronological age and MFI were similar. A strong decline with age among both C57BL/6J and J:DO training-set mice (Supp. Fig. S2E) was maintained when parameterized for frailty (Supp. Fig. S2F; R = 0.14; p-value = $8.6 * 10^{-4}$) and was also correlated with MFI (R = 0.21; p-value = $6.1 * 10^{-7}$).

Circadian regulation of movement decreased with age.

The multi-day, continuous-monitoring nature of our video data for DFI allowed us to uniquely incorporate circadian rhythm into our assessment of frailty. We implemented a measure of circadian behavior that contrasted the amount of observed movement between alternating twelve-hour blocks. This intentionally coincided with the alternating 12-hour light/dark periods experienced by the mice (see Methods), but we did not require strict adherence to the light cue. Rather, our implementation measured the overall periodicity of behavior. There are other methods for evaluating circadian rhythms (Shimomura et al, 2001), but this approach captures a known pathology of aging: the maintenance of activity during what should be a rest period (Musiek & Holtzman, 2016).

Circadian regulation of ambulatory activity was observed to lessen with age among both C57BL/6J and J:DO training-set mice, both on the wheel (Supp. Fig. S3A) and on the floor of the cage (Supp. Fig. S3B). When parameterized for frailty, the decline of circadian activity with age was maintained, both for wheel-running activity (Supp. Fig. S3C; R = 0.18; p-value = $2.1 * 10^{-5}$) and for movement on the floor of the cage (Supp. Fig. S3D; R = 0.11; p = $6.9 * 10^{-3}$). Both metrics also correlated with overall MFI, with correlation coefficients of 0.13 (p-value = $2.1 * 10^{-3}$) and 0.11 (p-value = 0.011) for wheel-based and cage floor-based activity, respectively.

Frailty measurements of coat quality, body-weight change, and nest movement showed no statistical change with age.

Three components of our DFI implementation seemed justifiable to include but nonetheless performed poorly against the chronological age and/or MFI values of our test set. The first of these sought to quantify the movement of nest material. It is known that mice in pain or distress will expend less effort constructing nests (Gaskill et al, 2013), a phenomenon that is most effectively observed when mice are first provided with nesting material (Giménez-Llort & Torres-Lista, 2021). Our DFI analysis was not timed to the specific time at which the mouse was introduced to a new cage with new nest material and was therefore limited to the continued maintenance and modification of existing nests. Frailty was therefore parameterized to only capture instances of severely depleted engagement with the nest and was therefore only sparsely observed (Supp. Fig. S4A). Though nest-moving frailty trended positively with both chronological age (R = 0.05) and MFI (R = 0.07), in neither case was the association statistically significant. This parameter had no obviously analogous component of MFI. The most highly correlated MFI component was "whisker loss" (R = 0.14; p-value = $9.3 * 10^{-4}$).

The second poorly-performing DFI component was "body weight delta". The foundation of this parameter was a body-weight prediction tool. First developed and tested on an independent data set to predict body weight for a mouse by averaging from estimates taken across a day's worth of video (sampled every ten minutes; results averaged across 144 estimates/day), this model produced daily estimates within ~4 grams of the laboratory measured body weights (R = 0.88 across a test-set of 250 measurements with an SD = 33.9 grams; p-value = $7.2 * 10^{-77}$; Supp. Fig. S4B). For frailty, this prediction tool was used to identify rapid gain or loss of weight by regressing across estimates taken once-per-minute across each designated DFI measurement period

Rapid weight gain or loss is a known harbinger of mortality in mice that is commonly used as a humane endpoint (Toth, 1997). It is sometimes included in traditional frailty assessments, but doing so requires the manual collection of weights at disparate time points, therefore modifying the nature of the frailty index into a longitudinal assessment (e.g. Mach et al, 2022). For DFI, the trend in body weight across the ~one-week interval of observation time was used to calculate body weight dynamics as a single-observation measurement. Among both C57BL/6J and J:DO training-set mice, this method identified body weights as being highly stable, with a limited number of outlier exceptions (Supp. Fig. S4C). For frailty, this metric was parameterized to capture those outliers. The resulting frailty scores were significantly correlated with neither

chronological age (Supp. Fig. S4D; R = -0.01; p-value = 0.89) nor MFI (R = -0.00; p-value = 1.00). The two most highly correlated individual MFI components were “body condition score” (R = 0.09; p-value = 0.025) and “vision loss” (R = 0.13; p-value = $2.2 * 10^{-3}$). The correlation with “body condition score” suggested that some appropriate signal was captured by this metric, but that signal was overwhelmed by the noise of the measurement. Reassuringly, MFI “body condition score” had a negative, non-significant correlation with chronological age in our test cohort (R = -0.04; p-value = 0.30), suggesting that the inability of this parameter to correlate with chronological age was not entirely due to inaccuracy of the measurement.

The third poorly-performing DFI component was “coat quality”. For this metric, the Sobel gradient estimator was used to quantify the roughness of the portion of images containing the mouse, as determined by a segmentation model (see Methods). Parameterized for frailty, no correlation was found with chronological age (Supp. Fig. S4E; R = -0.06; p-value = 0.19), and a negative correlation was found with MFI (R = -0.10; p-value = 0.018). This was in spite of some separation in a preliminary test set of images labeled as having “good” or “bad” coats, using this method (Supp. Fig. S4F). Conceptually, “coat quality” could have encompassed multiple components of the MFI: “alopecia”, “loss of fur color”, “dermatitis”, “coat condition”, and/or “piloerection”. All of these were expected to increase the visual unevenness of the mouse’s coat. However, only one of these five MFI components positively correlated with DFI “coat quality”, and it was the only one of the five with a statistically significant correlation: “loss of fur color” (R = 0.13; p-value = $2.7 * 10^{-3}$). That MFI value also had a negative correlation with chronological age in our frailty study, though that correlation was not statistically significant (R = -0.04; p-value = 0.32). Taken together, these results suggested that the Sobel estimate accurately captured the color-loss aspect of coat condition, and that supplementation with additional measurements to better capture other aspects of coat condition would be preferential to abandonment of this parameter.

Performance improves with removal of low-performing parameters

We investigated the extent to which the performance of DFI versus the age and MFI metrics improved if we left out the three under-performing components, creating an optimized DFI. Consistent with the idea that nest movement, body weight change, and coat condition all contributed orthogonal noise, the original and optimized DFI values were highly correlated (Pearson R = 0.93 for C57B/6J; Pearson R = 0.87 for J:DO). Unsurprisingly, this increased the correlations with those metrics: with age, Pearson R increased to 0.63 (p-value = $3.2 * 10^{-11}$) for C57B/6J and 0.36 (p-value = $2.2 * 10^{-18}$) for J:DO; with MFI, Pearson R increased to 0.57 (p-value = $5.2 * 10^{-9}$) for C57B/6J and 0.27 (p-value = $4.9 * 10^{-11}$) for J:DO.

These results are presented as a purely most-mortem analysis, since the decision to remove these three parameters was based on our final evaluation rather than a preliminary training or pre-validation test set.

References

Bradski, G. (2000). The OpenCV Library. *Dr. Dobb's Journal of Software Tools*.

Gaskill, B. N., Karas, A. Z., Garner, J. P., & Pritchett-Corning, K. R. (2013). Nest Building as an Indicator of Health and Welfare in Laboratory Mice. *Jove-Journal of Visualized Experiments*.

Giménez-Llort, L., & Torres-Lista, V. (2021). Social nesting, animal welfare, and disease monitoring. *Animals*, *11*(4), 1079.

Greenwood, B. N., & Fleshner, M. (2019). Voluntary wheel running: a useful rodent model for investigating mechanisms of stress robustness and exercise motivation. *Current opinion in behavioral sciences*, *28*, 78-84.

Howard, A. G., Zhu, M., Chen, B., Kalenichenko, D., Wang, W., Weyand, T., ... & Adam, H. (2017). Mobilenets: Efficient convolutional neural networks for mobile vision applications. *arXiv preprint arXiv:1704.04861*.

Liu, W., Anguelov, D., Erhan, D., Szegedy, C., Reed, S., Fu, C. Y., & Berg, A. C. (2016, October). Ssd: Single shot multibox detector. In *European conference on computer vision* (pp. 21-37). Springer, Cham.

Mach, J., Allore, H., Gnjidic, D., Gemikonakli, G., Kane, A. E., Howlett, S. E., ... & Hilmer, S. N. (2022). Preclinical frailty assessments: Phenotype and frailty index identify frailty in different mice and are variably affected by chronic medications. *Experimental gerontology*, 111700.

Musiek, E. S., & Holtzman, D. M. (2016). Mechanisms linking circadian clocks, sleep, and neurodegeneration. *Science*, *354*(6315), 1004-1008.

Shimomura, K., Low-Zeddies, S. S., King, D. P., Steeves, T. D., Whiteley, A., Kushla, J., ... & Takahashi, J. S. (2001). Genome-wide epistatic interaction analysis reveals complex genetic determinants of circadian behavior in mice. *Genome research*, *11*(6), 959-980.

Simonyan, K., & Zisserman, A. (2014). Very deep convolutional networks for large-scale image recognition. *arXiv preprint arXiv:1409.1556*.

Sobel, I., & Feldman, G. (1968). A 3x3 isotropic gradient operator for image processing. *a talk at the Stanford Artificial Project in*, 271-272.

Tan, M., & Le, Q. (2019, May). Efficientnet: Rethinking model scaling for convolutional neural networks. In *International conference on machine learning* (pp. 6105-6114). PMLR.

Toth, L. A. (1997). The moribund state as an experimental endpoint. *Journal of the American Association for Laboratory Animal Science*, *36*(3), 44-48.

Viterbi, A. (1967). Error bounds for convolutional codes and an asymptotically optimum decoding algorithm. *IEEE transactions on Information Theory*, *13*(2), 260-269.

Whitehead, J. C., Hildebrand, B. A., Sun, M., Rockwood, M. R., Rose, R. A., Rockwood, K., & Howlett, S. E. (2014). A clinical frailty index in aging mice: comparisons with frailty index data in humans. *Journals of Gerontology Series A: Biomedical Sciences and Medical Sciences*, *69*(6), 621-632.

Supplemental Figure Legends

Supplemental Figure S1: Experimental design and methods details.

A) A schematic diagram of the J:DO experimental design.
B) Performance of the wheel-spin detection pipeline versus test data. For each of four different cohorts of mice, 100 10-second video clips were manually annotated for the number of wheel spins that occurred, in increments of half-spins, based on appearance and disappearance of the black marker. Those values are correlated against the number of spins annotated by the ML pipeline across the same time segment. The four cohorts included footage of either C57BL/6J mice or young, medium-age, or old J:DO mice. See Methods for details.
C) The distributions of wheel-spin counts across 10-second clips from the four manually annotated test sets from panel (B).
D) Sobel values versus median pixel brightness for training-set J:DO mice, colored according to human assignment of coat color.

Supplemental Figure S2: DFI components based on ambulatory activity captured age-related decline.

A) Training set distributions and frailty parameterization for total wheel distance. Left: statistics for three consecutive, week-long measurements each, for three cohorts of differently-aged C57BL/6J mice. Right: similar statistics for single-week measurements from differently-aged J:DO mice. For each cohort, green bars indicate the mean value; dark blue bars indicate the median value; and light blue bars indicate the 10th and 90th percentiles. Far right: red arrows indicate the threshold values for a frailty score of zero (upper arrow) or one (lower value). Frailty values in between the thresholds (gray rectangle) were given linearly intermediate frailty values (see Methods).
B) Chronological age (x-axis) versus wheel distance frailty scores (y-axis) for the main study population. Mean (horizontal bars) and standard deviations (vertical bars) are shown for each of three measurements taken for each of eight birth cohorts (with cohort 8 split into two sub-groups: see Key from Main Fig. 3). The linear regression of age versus wheel distance frailty score, for all individual values, is shown in green.
C) Training set distributions and frailty parameterization for wheel gait speed. Plotted as in (A).
D) Chronological age (x-axis) versus wheel gait speed frailty scores (y-axis) for the main study population. Plotted as in (B).
E) Training set distributions and frailty parameterization for cage-floor gait speed. Plotted as in (A).
F) Chronological age (x-axis) versus cage-floor gait speed frailty scores (y-axis) for the main study population. Plotted as in (B).

<u>Supplemental Figure S3</u>: DFI components based on circadian activity captured age-related decline.

A) Training set distributions and frailty parameterization for the circadian ratio of wheel-running activity (see Methods). Plotted as described for Supp. Fig. 2A.
B) Training set distributions and frailty parameterization for the circadian ratio of movement on the floor of the cage (see Methods). Plotted as described forSupp. Fig. 2A.
C) Chronological age (x-axis) versus circadian wheel-running frailty scores (y-axis) for the main study population. Plotted as in Supp. Fig. 2B.
D) Chronological age (x-axis) versus frailty scores for circadian movement on the floor of the cage (y-axis) for the main study population. Plotted as in Supp. Fig. 2B.

<u>Supplemental Figure S4</u>: DFI components based on nest movement, rapid body weight change, and coat roughness failed to capture age-related decline.

A) Chronological age (x-axis) versus nest movement frailty scores (y-axis) for the main study population. Plotted as in Supp. Fig. 2B.
B) Performance of the body weight prediction model using 24-hour averages. Measured body weights (x-axis) versus predicted body weights based on statistical averaging of model outputs from across the same 24-hour period (y-axis) are plotted in green: each point is a measurement/prediction instance. Shown along with the calculated regression line (blue) and identity line (red).
C) Training set distributions and frailty parameterization for changes to body weight. Plotted as described for Supp. Fig. S2A, but with individual values outside of the 10th and 90th percentiles additionally plotted (purple), and with both positive and negative thresholds for the absolute-value-based frailty score depicted (inner thresholds correspond to zero; outer thresholds correspond to one; see Methods).
D) Chronological age (x-axis) versus frailty scores based on changes in body weight (y-axis) for the main study population. Plotted as in Supp. Fig. S2B.
E) Chronological age (x-axis) versus frailty scores based on coat roughness (y-axis) for the main study population. Plotted as in Supp. Fig. S2B.
F) A histogram of Sobel scores (see Methods) for images from a preliminary test set, labeled as having “good” or “bad” coats.

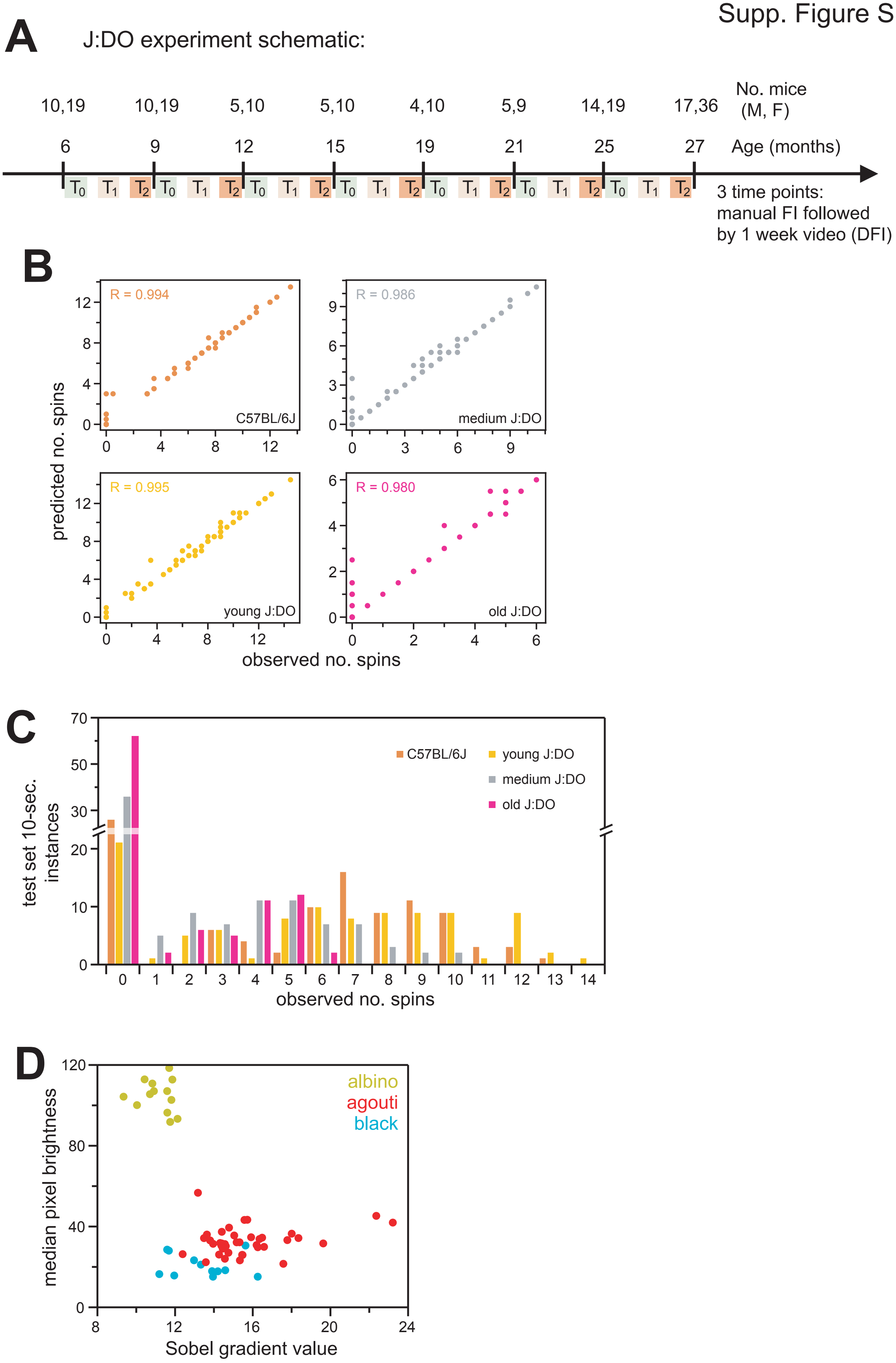
A
J:DO experiment schematic:
No. mice (M, F)
10,19 10,19 5,10 5,10 4,10 5,9 14,19 17,36
Age (months)
6 9 12 15 19 21 25 27
T0 T1 T2
3 time points: manual FI followed by 1 week video (DFI)
B
predicted no. spins
observed no. spins
R = 0.994
C57BL/6J
R = 0.986
medium J:DO
R = 0.995
young J:DO
R = 0.980
old J:DO
C
test set 10-sec. instances
observed no. spins
C57BL/6J
young J:DO
medium J:DO
old J:DO
D
median pixel brightness
Sobel gradient value
albino
agouti
black

Supp. Figure S2

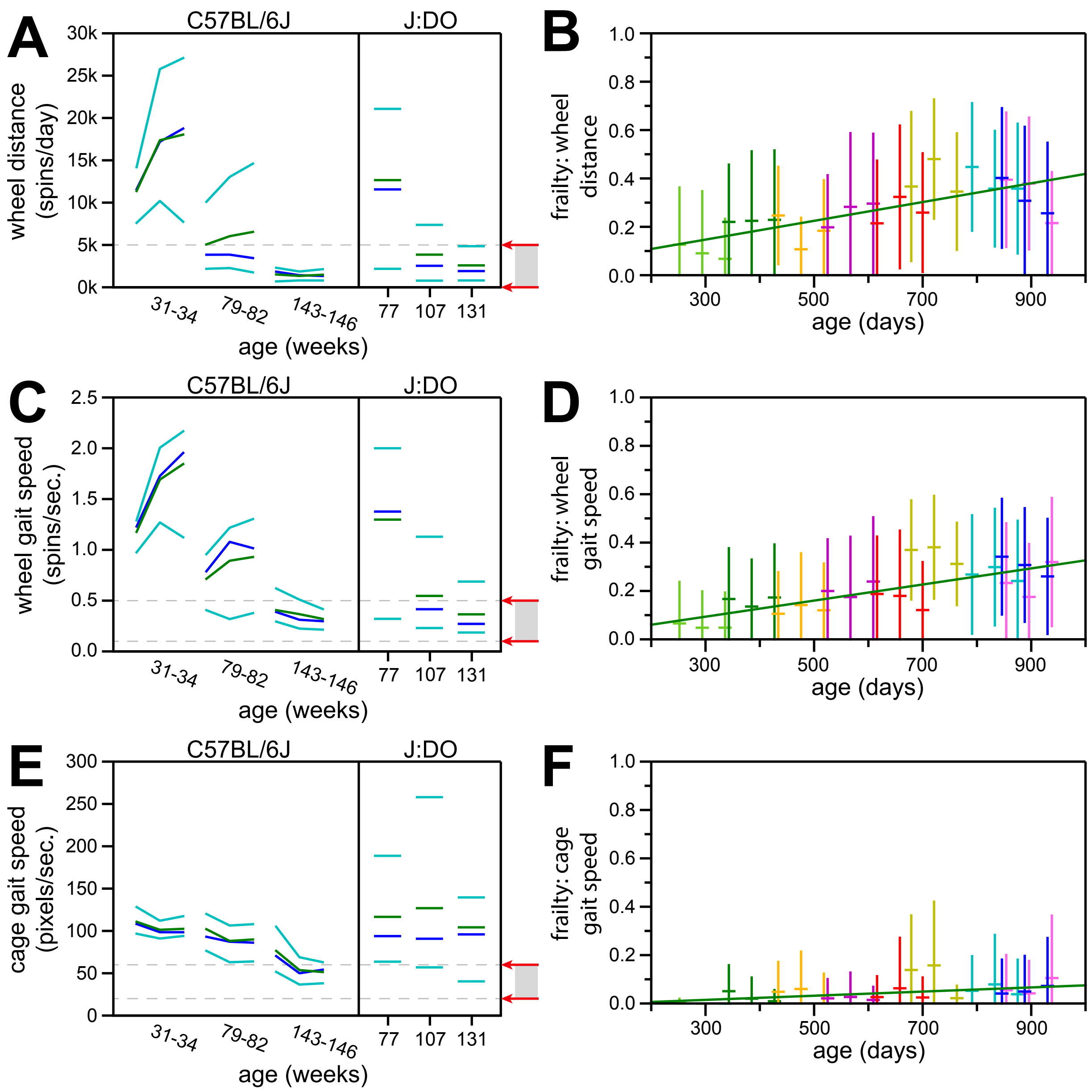

Supp. Figure S3

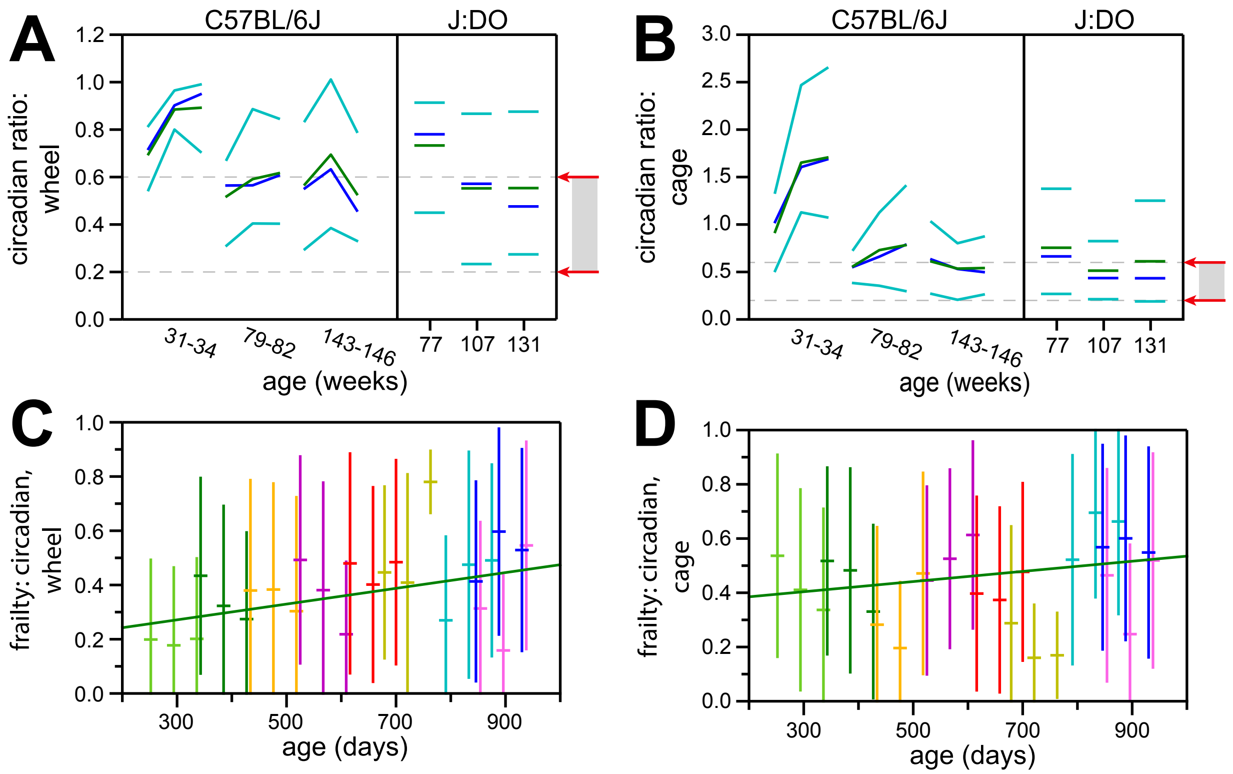

Supp. Figure S4

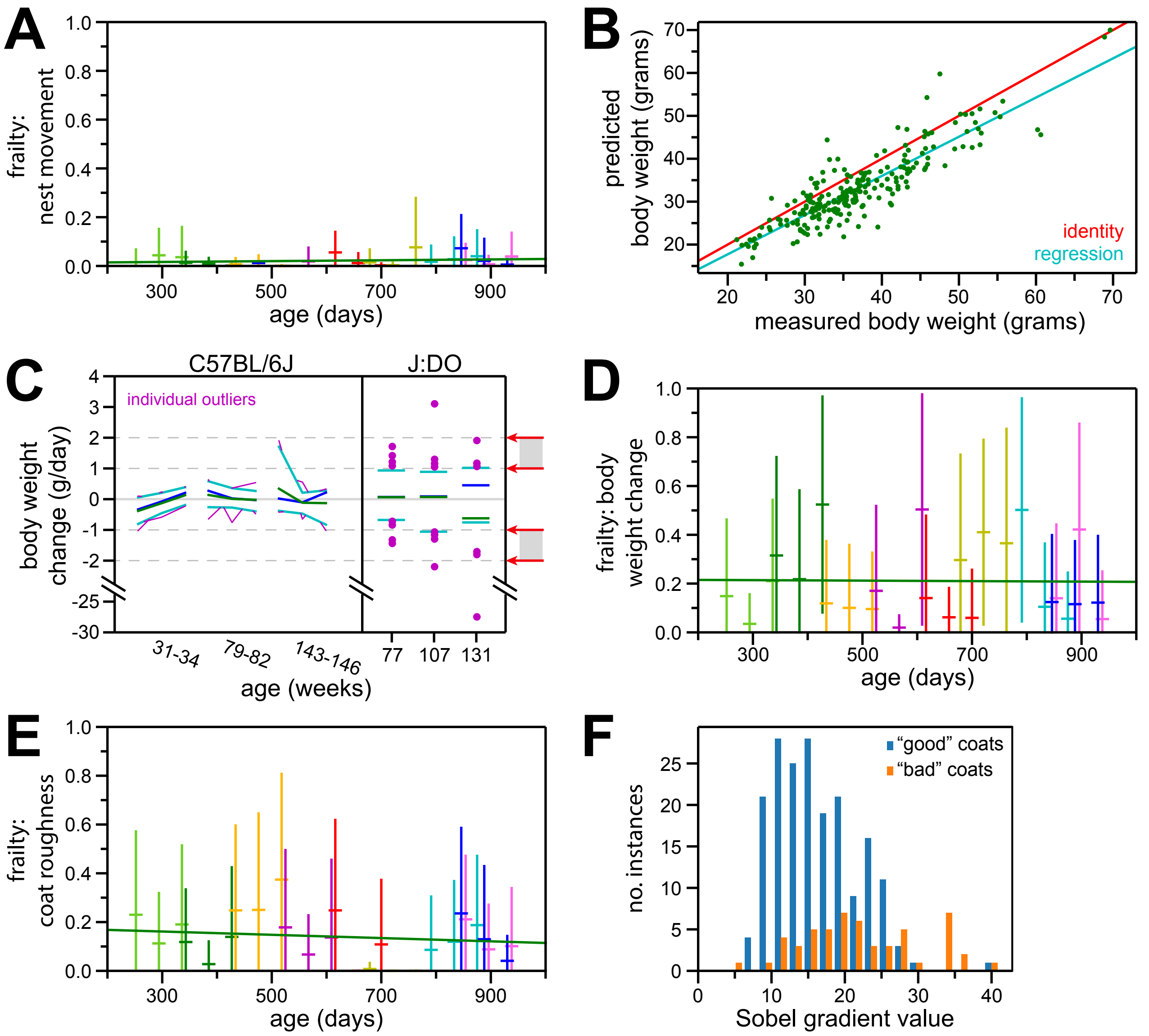

Supplement: glad035_suppl_Supplementary_Materials [file glad035_suppl_supplementary_materials.zip › glad035_suppl_Supplementary_Data.pdf]
