# Supplementary material for: An Automated, Home-Cage, Video Monitoring-based Mouse Frailty Index Detects Age-associated Morbidity in C57BL/6 and Diversity Outbred Mice
Source: J Gerontol A Biol Sci Med Sci. 2023 Jan 27;78(5):762–70. doi: 10.1093/gerona/glad035 (PMC10172975; doi:10.1093/gerona/glad035)
Supplement: glad035_suppl_Supplementary_Materials [file glad035_suppl_supplementary_materials.zip › glad035_suppl_Supplementary_Table_Legends.docx]

Supplementary Table 1. Census and MFI score data for C57B/6J mice.

Supplementary Table 2. Census data on all J:DO mice obtained for this study.

Supplemental Table 3: MFI score data for J:DO mice.

Supplementary Table 4. DFI score data for C57B/6J mice: overall score and parameterized components.

Supplementary Table 5. DFI score data for J:DO mice: overall score and parameterized components.

Supplementary Table 6. Pre-parameterized values for DFI components, for C57B/6J mice.

Supplementary Table 7. Pre-parameterized values for DFI components, for J:DO mice.

Supplementary Table 8. Time intervals of DFI video collection for J:DO mice, compliant with DFI software.

Supplementary Table 9. Qualifying MFI/DFI pairs for J:DO mice.

Supplementary Table 10. numerical values for all statistics plotted in Figs. 3 and 4.

Supplementary Text: Results, Methods, and four figures (Supp. Figs. 1-4).
